# Improved catalytic performance and molecular insight for lipoxygenase from *Enterovibrio norvegicus* via directed evolution

**DOI:** 10.3389/fbioe.2023.1305582

**Published:** 2023-11-16

**Authors:** Bingjie Zhang, Huibing Chi, Juan Shen, Yang Tao, Zhaoxin Lu, Fengxia Lu, Ping Zhu

**Affiliations:** College of Food Science and Technology, Nanjing Agricultural University, Nanjing, China

**Keywords:** *Enterovibrio norvegicus*, lipoxygenase, directed evolution, catalytic activity, molecular dynamic simulation, binding free energy, structure analysis

## Abstract

Lipoxygenase (LOX) holds significant promise for food and pharmaceutical industries. However, albeit its application has been hampered by low catalytic activity and suboptimal thermostability. To address the drawbacks, a directed evolution strategy was explored to enhance the catalytic activity and thermostability of LOX from *Enterovibrio norvegicus* (EnLOX) for the first time. After two rounds of error-prone polymerase chain reaction (error-prone PCR) and one generations of sequential DNA shuffling, all of four different mutants showed a significant increase in the specific activity of EnLOX, ranging from 132.07 ± 9.34 to 330.17 ± 18.54 U/mg. Among these mutants, D95E/T99A/A121H/S142N/N444W/S613G (EAHNWG) exhibited the highest specific activity, which was 8.25-fold higher than the wild-type enzyme (WT). Meanwhile, the catalytic efficiency (*K*_*cat*_ */K*_*m*_) of EAHNWG was also improved, which was 13.61 ± 1.67 s^−1^ μM^−1^, in comparison to that of WT (4.83 ± 0.38 s^−1^ μM^−1^). In addition, mutant EAHNWG had a satisfied thermostability with the *t*_1/2,50 °C_ value of 6.44 ± 0.24 h, which was 0.4 h longer than that of the WT. Furthermore, the molecular dynamics simulation and structural analysis demonstrated that the reduction of hydrogen bonds number, the enhancement of hydrophobic interactions in the catalytic pocket, and the improvement of flexibility of the lid domain facilitated structural stability and the strength of substrate binding capacity for improved thermal stability and catalytic efficiency of mutant LOX after directed evolution. Overall, these results could provide the guidance for further enzymatic modification of LOX with high catalytic performance for industrial application.

## 1 Introduction

Lipoxygenase (LOX) functions as a non-heme iron oxidoreductase, facilitating the catalysis of polyunsaturated fatty acids (PUFAs) containing 1-*cis*, 4-*cis*-pentadiene units into fatty acid hydroperoxides (Sugio et al., 2018). To date, LOX has garnered attention due to its extensive potential across various sectors, including the food, chemical, and pharmaceutical industries (Stolterfoht et al., 2019). Its applications encompass the bleaching of colored components in food, paper, and textile processes, as well as the modification of lipids from diverse raw materials to yield oleochemicals and aroma compounds (Haeggstrom and Funk, 2011; Qian et al., 2017). Additionally, LOX exhibits the capability to enhance the cross-linking of gluten, consequently improving dough rheology—a quality essential for reinforcing wheat dough (Zhang et al., 2016; Shi et al., 2020). However, despite its promising utility as a biocatalyst, LOX encounters challenges, including low catalytic efficiency, inadequate thermal stability, and susceptibility to acids and alkalis, which significantly impede its widespread utilization (Casey and Hughes, 2004; Heshof et al., 2016; Oroz et al., 2016). Consequently, there exists a pressing need for the development of high-performance LOX mutants to meet the demands of industrial applications.

In recent years, protein engineering has proven to be a potent approach for engineering natural enzymes to gain valuable properties for industrial applications, rendering them valuable for industrial applications (Zhou J. Y. et al., 2022). To address the limitations of LOX, various enzyme engineering techniques have been employed, including loop modification (Kalms et al., 2017), site-directed mutation (Diao et al., 2016; Kalms et al., 2017), and the utilization of self-assembling amphiphilic peptides (Lu et al., 2013), which all aimed at enhancing the enzyme’s catalytic activity and thermostability. Although some of these approaches have indeed shown promise in enhancing LOX’s performance, the simultaneous improvement of both thermostability and catalytic activity remains a challenging endeavor. Directed evolution, as a classical and efficient method in protein engineering, involves highly randomized mutation sites, making it versatile in enhancing a broad spectrum of enzyme properties (Wang H. Y. et al., 2020), including catalytic activity, thermal stability, and substrate specificity (Lu et al., 2019; Xiong et al., 2021; Yu et al., 2022). Directed evolution has proven to be a potent tool in enhancing the catalytic activity of enzymes (Lu et al., 2019; Xiong et al., 2021; Yang et al., 2022). Researchers have successfully improved the catalytic activity of various enzymes, such as α-amylase (Pan et al., 2020), phytase (Besirlioglu et al., 2017), and cyclodextrin glucosyltransferase (Melzer et al., 2016), through directed evolution. However, it is worth noting that there have been relatively few reports on the directed evolution of LOX (Guo et al., 2014). Since the quest for a heat-resistant, acid/alkali-resistant LOX with efficient catalytic activity suitable for industrial production remains unfulfilled, directed evolution is of great significance for the pursuit of LOX mutants with exceptional performance.

In the previous study, a thermo- and pH-stable LOX derived from *Enterovibrio norvegicus* DSM 15893 (EnLOX) was successfully generated through heterologous expression (Zhang et al., 2022). However, the catalytic activity of EnLOX towards linoleic acid (LA) was measured at 40.02 ± 3.31 U/mg, suggesting ample room for improvement. Herein, random mutations were introduced into the gene of EnLOX via *error-prone* PCR and *DNA shuffling*. Mutants exhibiting high catalytic activity were subsequently identified through high-throughput screening. Concurrently, molecular dynamics (MD) simulations and structural analysis were conducted for both wild-type and mutant EnLOXs to provide insights into the molecular underpinnings of the catalytic mechanism. The satisfactory thermostability and heightened catalytic efficiency observed in the EnLOX mutant underscore its potential value in industrial applications.

## 2 Materials and methods

### 2.1 Materials

The LOX gene from *E. norvegicus* DSM 15893, encoded in plasmid pET-28a (+)-EnLOX (Zhang et al., 2022), has been maintained within the laboratory. The vector pET-28a (+) and *Escherichia coli* BL21 (DE3) were sourced from Novozymes Co., Ltd. (Wilmington, United States). Essential kits and reagents, including the FastPure Plasmid Mini Kit, FastPure Gel DNA Extraction Mini Kit, ClonExpress II One Step Cloning Kit, and 2×Taq Master Mix (Dye Plus), were procured from Vazyme Co., Ltd. (Nanjing, China). MnSO_4_, MgCl_2_ and Super Pfu DNA polymerase were purchased from Takara Biotechnology Co., Ltd. (Dalian, China). Kanamycin and isopropyl-β-_D_-thiogalactopyranoside were obtained from Solarbio Co., Ltd. (Beijing, China). Linoleic acid (LA) was sourced from Sigma Co., Ltd. (Steinheim, Germany). All other chemicals employed in the study were analytical grade.

### 2.2 Error-prone PCR and the recombinant library construction

The template for the first round of *error-prone* PCR was plasmid pET-28a (+)-EnLOX, containing 2,190 bp native LOX gene. Plasmid-derived primers F (5′-gtg​ccg​cgc​ggc​agc​cat​atgCAT​ATGCAT​CGT​AAG​AAA​ACC​CC-3′) and R (5′-gtg​gtg​gtg​gtg​gtg​ctc​gagCTC​GAGTTA​GAT​GCT​AAT​GCT​ATT​CG-3′) were designed with embedded *Nde I* and *Xho I* restriction sites (underlined) for subsequent amplification.

The genes comprising the first-generation mutant libraries were prepared by the *error-prone* PCR method, which contained 50 ng of plasmid pET 28a-EnLOX as the template, 0.4 μM each primer, 0.5 mM dCTP, 0.5 mM dTTP, 9 mM Mg^2+^, 0.75 mM Mn^2+^ and 25 μL 2×Taq PCR Mix (Lu et al., 2019). The plasmid fragments were amplified by a Veriti PCR (Shanghai, China). Mutant plasmids containing all beneficial mutations from the first round of *error-prone* PCR were used as templates to prepare the second round of *error-prone* PCR libraries. The PCR products were recovered using FastPure Gel DNA Extraction Mini Kit. The purified products were digested with *Nde I* and *Xho I,* and ligated into the expression vector pET-28a(+) by the ClonExpress II One Step Cloning Kit. The ligation products were transformed into *E. coli* BL21(DE3) for constructing the high-throughput screening library. The mutants screened were sequenced by Sangon Biotech Co., Ltd. (Shanghai, China).

### 2.3 DNA shuffling and the recombinant library construction

Three mutants (32-12F, 28-2A, 13-6H) displaying higher enzyme activity, which were identified from the aforementioned mutant library created via *error-prone* PCR, served as templates for *DNA shuffling* (Guo et al., 2014). These mutant EnLOX genes were amalgamated in equal proportions, followed by cloning and subsequent digestion with DNase I at 37°C for 3 min. To halt the digestion reaction, the mixture was heated to 90°C for 10 min. The extent of DNA fragmentation was assessed through 2% agarose gel electrophoresis, and small-sized fragments (50–100 bp) were isolated. These fragments were reassembled using a primer-free PCR approach, with the reaction system comprising 25 μL of 50–100 bp fragments (at a final concentration of 10 ng/mL) and 25 μL of 2 × Taq PCR Mixture buffer. The primer-free PCR reaction conditions mirrored those of *error-prone* PCR, except that the number of intermediate cycles was set to 40. The full-length mutant EnLOX genes were subsequently amplified through PCR, employing the same primers and conditions as previously mentioned. After amplification, the mutant EnLOX genes were ligated into the linearized pET-28a(+) expression vector (Zhang et al., 2022). The resulting recombinant products were introduced into *E. coli* BL21(DE3) through chemical transformation to generate the *DNA shuffling* library.

### 2.4 High-throughput screening of mutant library

A method previously described by Guo et al. was adopted with certain modifications (Guo et al., 2014). All colonies were individually selected and transferred into 96-well plates, each containing 600 μL of Luria-Bertani (LB) medium supplemented with 50 μg/mL kanamycin. As a positive control in these experiments, an *E. coli* BL21 (DE3) clone harboring pET-28a (+)-EnLOX was employed. Cultures were initiated by inoculating 50 μL of overnight cultures into 600 μL of LB-kanamycin medium in fresh 96 deep-well plates. These cultures were then incubated at 37°C and 200 rpm for 3 h. Subsequently, isopropyl-β-_D_-thiogalactopyranoside was added into each well with a final concentration of 100 μg mL^−1^, and inducing at 16°C for another 16 h. The cells were harvested by centrifugation at 4,000 × g for 5 min. The harvested cells from each well were resuspended in 100 μL of lysis buffer (composed of 50 mM Tris-HCl and 300 mM NaCl at pH 7.5). The plates were subjected to a water bath at 37°C for 20 min, followed by freezing at −80°C for 120 min and subsequent thawing in water bath at 37°C for 20 min. This freeze/thaw cycle was repeated for three times. The supernatant obtained after centrifugation at 4,000 × g for 5 min represented the crude enzyme. The high-throughput screening was conducted according to the potassium iodide-starch method with slight modifications (Iassonova et al., 2008). Firstly, 80 μL of 50 mM Tris-HCl buffer (pH 8.0), 10 μL of enzyme and 10 μL of 1.73 mmol/L LA were mixed at 50°C for 4 min. Then, 60 μL of potassium iodide-starch solution and 40 μL of acetic acid solution (40%) were added to each well, and placed at room temperature for 8 min. Furtherly, the activities of the enzyme were assayed by a BioTek Synergy H1 (Vermont, United States) in the absorbance at 470 nm. One international unit of LOX activity was defined as the increase at 470 nm of 0.001 per min. Mutants exhibiting high enzymatic activity were further screened using shake-flask preparations.

### 2.5 Expression and purification

The recombinant *E. coli* strains containing either the wild-type EnLOX (WT) or its mutant genes were selected and induced. The cells were collected by centrifugation (8,000 × g, 5 min), and resuspended in Tris-HCl buffer (0.05 mol/L Tris-HCl, 0.3 mol/L NaCl, pH 7.5). After cells were completely broken by D-3L High Pressure Homogenizer (PhD Technology LLC, United States), cell debris were removed by centrifugation (4°C, 10,000 × g, 30 min) (Zhang et al., 2022). The crude enzyme solutions were purified by the Ni-chelating affinity chromatography. The concentrations of the purified enzymes were determined by the Bradford method (Sharma and Tihon, 1988). The sodium dodecyl sulfate polyacrylamide gel electrophoresis (SDS-PAGE) was used to analyze the purity integrity, and molecular mass weight of the enzymes (Hui et al., 2017).

### 2.6 Enzymatic activity assays

The activities of both the EnLOX wild-type and its mutant were measured spectrophotometrically using LA as a substrate, following previously established methods (Zhang et al., 2022). The activity was assayed in 20 mmol/L Tris-HCl buffer (pH 8.0) at 50°C by monitoring the increase in absorbance at 234 nm, and the substrate was added to a 3 mL reaction system, making the final concentration of LA being 1.73 mmol/L. One unit of activity was defined as the amount of enzyme required to synthesize 1 mmol hydroperoxide per min with an extinction coefficient of 25,000 L/(mol cm) (Qian et al., 2018; Qi et al., 2020).

### 2.7 Biochemical characterization of recombinant enzymes

The kinetic parameters of the purified enzymes were measured with the final concentration of LA ranging from 10 to 300 mM at 50°C in 20 mM Tris-HCl buffer (pH 8.0). The kinetic data were fitted with the Michaelis-Menten equation using GraphPad software (Santiago, United States). Michaelis-Menten equation is shown as follows (Goličnik, 2011; Leow and Chan, 2019):

$$v_{0}=\frac{V_{\max}\left[S\right]}{K_{m}+\left[S\right]}$$

where $v_{0}$ , $V_{\max}$ , $\left[S\right]$ , and $K_{m}$ represent the initial rate, the maximum velocity of reaction, the substrate concentration, and the Michaelis constant, respectively.

The optimal reaction pH of these enzymes was measured at 50°C in the buffers of pH 3.0–10.0. The highest activity of each mutant was setted as 100%, so as to calculate the relative activities at each pH level. To evaluate the stability of enzyme at different pH levels, these enzymes were preincubated at 4°C for 10.0 h in buffers with varied pH levels. The original activity was defined as a 100% activity and the residual activity were assayed at 50°C in 20 mM Tris-HCl buffer (pH 8.0).

The optimal reaction temperature of these enzymes was measured in 20 mM Tris-HCl buffer (pH 8.0) at different temperatures (20°C–80°C). The maximum activity of each mutant within the aforementioned range was set as 100%, so as to calculate the relative activities at different temperatures. To estimate the thermostability of EnLOX wild-type and the mutant, the half-life (*t*_1/2_) of the enzymes were determined by measuring the residual enzyme activity after incubating at 50°C–70°C for 9 h, and the catalytic activity without heat treatment was defined as 100%.

### 2.8 Analysis of circular dichroism spectroscopy

Circular dichroism spectroscopy analysis (CD) was performed to determine the changes in the secondary structure between EnLOX wild-type and its mutant EAHNWG. The enzyme samples were dissolved in 50 mM Tris-HCl buffer (pH 7.5) at a final concentration of 100 μg/mL, and the scanning was carried out in the wavelength range of 190–250 nm. The spectrum of the buffer blank was subtracted. The percentage of secondary structures of enzymes were estimated by DichroWeb (Whitmore and Wallace, 2008).

### 2.9 Molecular modeling

In order to explore the role of randomly introduced mutations in improving enzyme activity, the SWISS-MODEL was used to construct the three-dimensional (3D) models of EnLOX using *Pseudomonas aeruginosa* LOX (PDB ID: 4rpe) as template (Bordoli et al., 2009). Subsequently, the structure model of the mutant EAHNWG was created by Coot based on the structural model of EnLOX (Emsley and Cowtan, 2004). Furthermore, PyMOL was used for the visualization and analysis of the 3D structure of the molecules (Lill and Danielson, 2011).

### 2.10 Molecular docking and molecular dynamics (MD) simulation

To analyze the effect of mutation on the catalytic activity of EnLOX, the docking of ligand LA to both the wild-type EnLOX and the mutant EAHNWG was conducted using AutoDock Vina 1.1.2. Subsequently, the MD simulations of these enzyme-ligand complexes were carried out by Amber 18. The protein-ligand complexes were subjected to the Amber ff14SB force field. Hydrogen atoms were added to the system using the LEaP module, and a truncated octahedral TIP3P solvent cartridge was introduced at a distance of 10 Å from the system. To balance the system’s charge, Na^+^/Cl^−^ ions were added. The system energy was minimized through 2,500 steps of the steepest descent and 2,500 steps of conjugate gradient. The system was equilibrated for 100 ps under constant volume (NVT) and constant pressure (NPT) ensembles. Eventually, the MD simulations were performed for 50 ns at isobaric-isothermal (NPT) ensembles at 330 K.

Various parameters, including Root mean square deviation (*RMSD*), root mean square fluctuation (*RMSF*), hydrogen bond number, radius of gyration (*Rg*), solvent accessible surface area (*SASA*) were calculated based on the MD results. Additionally, the binding free energy between protein and ligand was determined by the MM/GBSA method (Genheden and Ryde, 2015). Lastly, the binding pattern of EnLOX-LA complexes were analyzed.

### 2.11 Statistical analysis

All the tests were performed in triplicate, and data were expressed as mean ± SD. SPSS 21.0 were used to analyze the differences between the variables. Statistical differences in correlation analysis and linear regression analysis were determined by one-way analysis of variance (ANOVA), and Duncan’s test and the significance level of *p* < 0.05 were used.

## 3 Results

### 3.1 Library construction and high-throughput screening of EnLOX mutants

**FIGURE 1:**
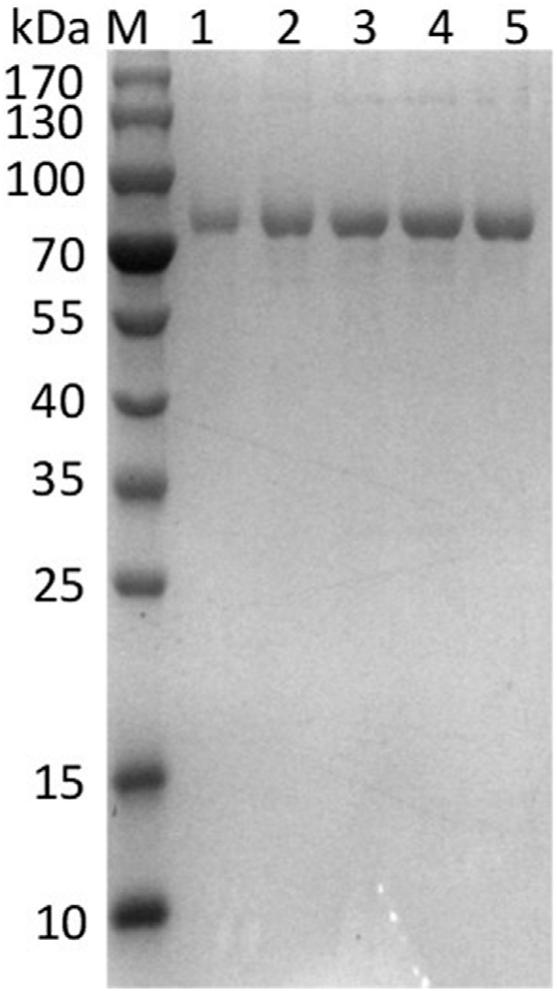
SDS-PAGE electrophoresis analysis of EnLOX wild-type and its mutants after purification. M: Protein marker; Lane 1: EnLOX wild-type; Lane 2-5: mutant 32-12F, 28-2A, 13-6H, and EAHNWG.

To pursue mutants displaying enhanced catalytic activity, the directed evolution of EnLOX was conducted. Initially, a mutant library comprising approximately 14,080 colonies was generated through two rounds of *error-prone* PCR. From this library, three promising candidates were identified and employed as templates for commencing the *DNA shuffling* experiments. Subsequently, through one successive rounds of *DNA shuffling*, four positively selected mutant with significantly improved activity were obtained from a new mutant library containing roughly 4,800 colonies. All mutants exhibited the molecular weight similar to that of the wild-type, approximately 80 kDa, implying their successful expression and homogeneous purification (Figure 1). Table 1 detailed the specific activity, nucleotide changes, and amino acid substitutions of the three mutants from *error-prone* PCR and the mutant derived from *DNA shuffling*. Among these mutants, EAHNWG (with six mutation sites) exhibited catalytic activities that were 8.25 times higher than that of the wild-type EnLOX. Notably, the specific activity of EAHNWG was measured at 330.17 ± 18.54 U/mg, which was significantly higher than that of the wild-type EnLOX. The specific activity of the mutant EAHNWG surpassed that of most previously reported LOXs, including *Calothrix* LOX (73.1 U/mg) (Qi et al., 2020), *Agrocybe aegerita* LOX (51.34 U/mg) (Karrer and Ruhl, 2019), *Pleurotus* LOX (130.3 U/mg) (Kuribayashi et al., 2002), and *P. aeruginosa* LOX (28.5 U/mg) (Lu et al., 2013).

**TABLE 1:** The enzymatic activity of a wild-type EnLOX and mutants obtained after the *error-prone* PCR and *DNA shuffling* enhancement.

Enzymes	Specific activities (U/mg)	Times	Nucleotide substitutions	Amino acid substitutions
WT	40.02 ± 3.31^e^	1.00	-	-
32-12F	195.30 ± 14.23^b^	4.88	G147A,A295G,C366T,A903G,A1333T,A1334G,C1335G,C1710G,A1840G	A49T,T99A,A121H,N444W,S613G
28-2A	162.08 ± 14.57^c^	4.05	T288A,A295G,C366T,A780C,A901G,A903G,G1338A,C1710G	D95E,T99A,A121H,E260A,K300E,G446S
13-6H	132.07 ± 9.34^d^	3.30	T87C,A295G,C366T,G428A,A570C,A903G,G1429A,C1710G	S29P,T99A,A121H,S142N,E190A,A476T
F-12E	330.17 ± 18.54^a^	8.25	T288A,A295G,C366T,G428A,A903G,A1333T,A1334G,C1335G,C1710G,A1840G	D95E,T99A,A121H,S142N,N444W,S613G

F-12E is named EAHNWG., The data were presented as means ± standard deviation (*n* = 3).

### 3.2 Enzymatic characterization of wild-type and mutant EnLOX

**FIGURE 2:**
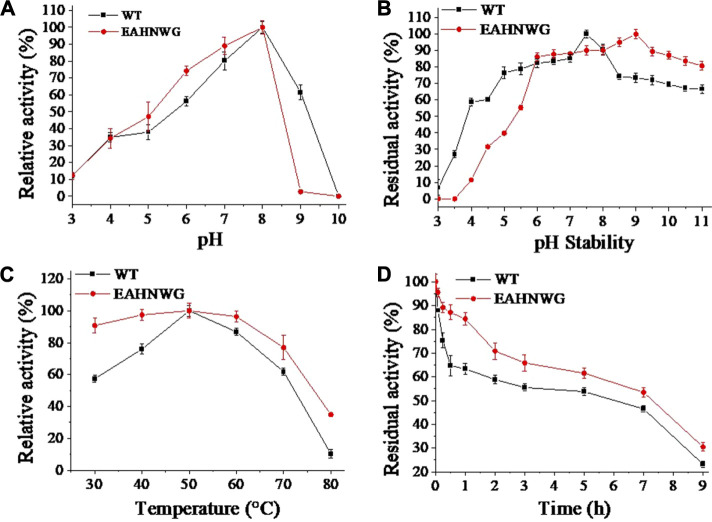
The effect of pH and temperature on enzymatic activity. **(A)** The relative activity of wild-type EnLOX and its mutant EAHNWG at various pH values; **(B)** The residual activity of wild-type EnLOX and mutant EAHNWG after incubation in different buffer preparations for 10 h at 4°C; **(C)** The relative activity of the wild-type EnLOX and mutant EAHNWG at different temperatures in 20 mM Tris-HCl buffer (pH 7.5); **(D)** The residual activity of wild-type EnLOX and mutant EAHNWG was measured after incubated at 50°C in 20 mM Tris-HCl buffer (pH 7.5) for different times (0–9 h); Data are expressed as the mean value (SD) of three independent experiments.

Considering the industrial applications for LOX, the enzymatic properties are essential characteristic for LOX mutant. The optimal pH for the catalysis by the mutant EAHNWG remained the same as that of the WT, being pH 8.0 (Figure 2A). After a 10 h incubation at 4°C, the mutant EAHNWG retained over 55% of its residual activity within the pH range of 5.5–10.5 (Figure 2B). This retention was higher than that of the WT, indicating its tolerance to weak acid and weak alkaline environment. This particular trait of mutant EAHNWG, with increased stability under varied pH conditions, might make it more adaptable to the complex food processing environments.

The optimal temperature for the catalysis by the EAHNWG was 50°C, which was the same as the WT, and the mutant EAHNWG showed higher catalytic activity than WT at 30°C–80°C (Figure 2C). To analyze the thermostability of the enzymes, the purified WT and mutant EAHNWG were incubated at 50°C (Figure 2D) for different times (0–9 h). Compared to WT, the *t*_*1/2*_ value of mutants EAHNWG at 50°C were extended approximately 0.4 h (Table 2). The result reveals that the mutant EAHNWG possesses a better thermostability than WT. Moreover, it is noted that the mutant EAHNWG showed a remarkable improvement in the optimal reaction temperature and excellent thermostability compared to WT. As a result, the mutant EAHNWG can be potentially applied in flour product and baking industries.

**TABLE 2:** Kinetics parameters, and half-life (*t*_1/2_) at 50°C of wild-type EnLOX and its mutant EAHNWG.

Enzymes	*K*_*m*_ (μmol/L)	*K*_*cat*_ (1/s)	*K*_*cat*_ */K*_*m*_ (L/μmol s)	*t*_1/2,50 °C_ (h)
WT	3.49 ± 0.21	16.86 ± 0.22	4.83 ± 0.38	6.04 ± 0.14
EAHNWG	2.36 ± 0.14	32.11 ± 0.27	13.61 ± 1.67	6.44 ± 0.24

The data were presented as means ± standard deviation (*n* = 3).

The kinetics of the enzymatic reaction play a crucial role in characterizing the catalytic properties of the enzyme. The *K*_*m*_ value of EAHNWG was found to be 32.38% lower (*p* < 0.05) than that of the WT, resulting in a 2.82-fold increase (*p* < 0.05) in catalytic efficiency (*K*_*cat*_ */K*_*m*_) (Table 2). This enhancement in catalytic efficiency and substrate affinity for the mutant enzymes is of significant importance. Notably, the *K*_*m*_ value for mutant EAHNWG was lower than that reported for LOXs from *Anabaena* (3.54 mM) (Diao et al., 2016), *Calothrix* LOX (6.70 μM) (Qi et al., 2020), and *P. aeruginosa* (6.40 mM) (Lu et al., 2013). This observation suggested that EAHNWG could meet the *K*_*m*_ requirements for industrial applications. These improved catalytic properties, coupled with enhanced substrate affinity, highlighted the potential industrial applications of the mutant enzyme (Chi et al., 2023). Besides, further details regarding the kinetic parameters of different LOXs towards LA (Supplementary Table S1), aligned with the substrate’s kinetic requirements.

### 3.3 Structure stability analysis of wild-type and mutant EnLOX

The enhanced enzyme activity in mutant EAHNWG was speculated to arise from structural variations compared to the WT. Circular dichroism, a technique capable of assessing alterations in protein secondary structure, was employed to calculate and analyze the proportions of secondary structures in both the mutant and wild-type enzymes (Supplementary Table S2). Notably, the percentage of α-helices in mutant EAHNWG increased from 26.9% in the WT to 34.6%, while the proportion of random coils decreased from 34.3% in the WT to 29.8%. Studies have indicated that an increased percentage of random coils can enhance the overall protein structure’s flexibility. These findings initially supported the idea that the elevated α-helix ratio could contribute to enhanced enzyme stability. Furthermore, the increased content of random coils in mutant EAHNWG contributed to greater structural flexibility, likely affecting regions near the catalytic active sites and the lid structure, rendering them more flexible and mobile. Consequently, substrate accessibility to the active sites was enhanced, leading to improved catalytic efficiency. This structural insight provided a plausible explanation for the observed increase in enzyme activity.

**FIGURE 3:**
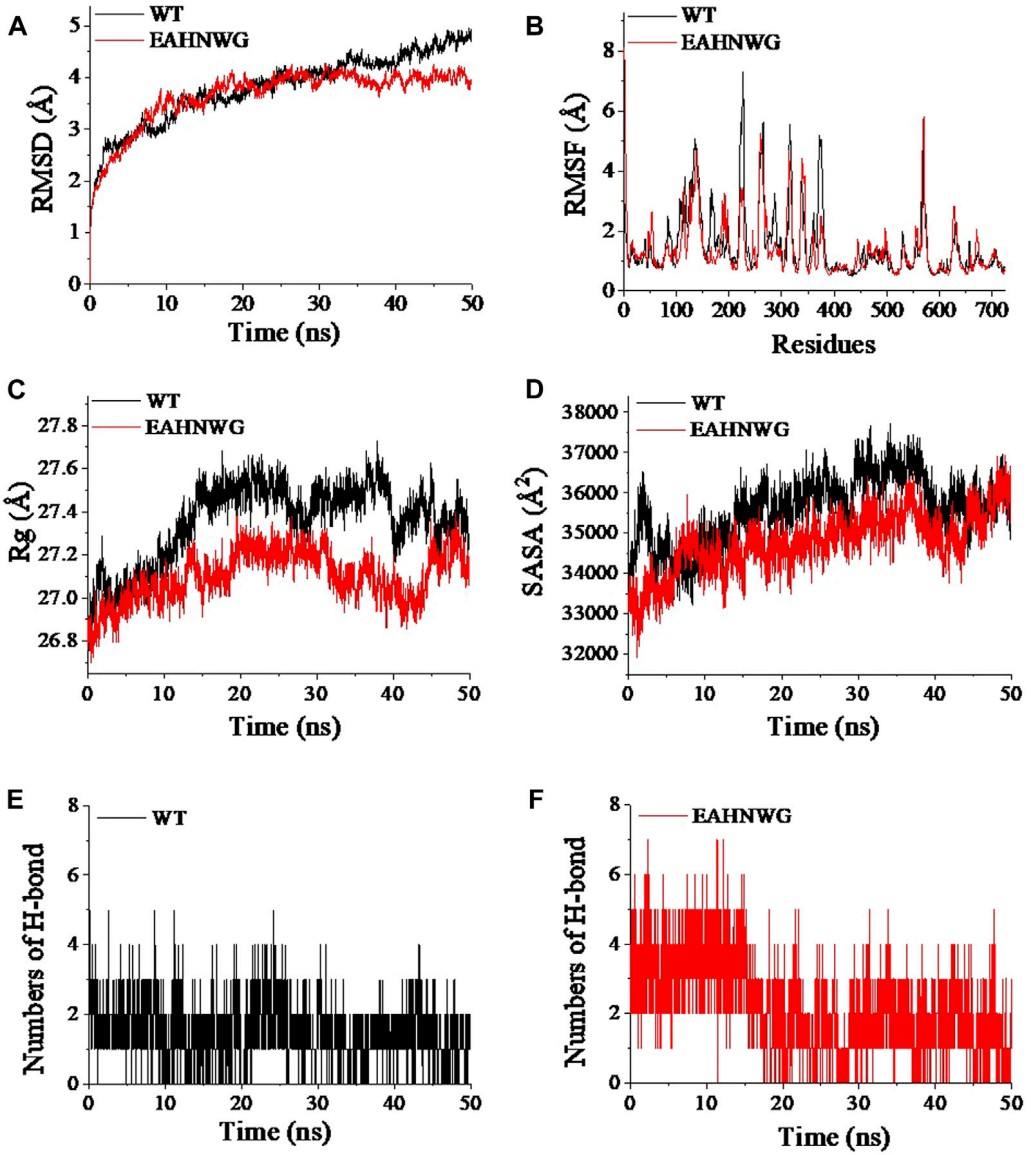
RMSD, RMSF, Rg, numbers of hydrogen bond, and SASA values of wild-type EnLOX and mutant EAHNWG. **(A)** RMSD; **(B)** RMSF; **(C)** Rg; **(D)** SASA; **(E)** Number changes of hydrogen bonds between LA and WT; **(F)** Number changes of hydrogen bonds between LA and EAHNWG.

In addition, the impact of LA binding on the structural stability of wild-type EnLOX and mutant EAHNWG during MD simulations was subjected to analysis. *RMSD* values from the MD simulations were employed to gauge the complex’s motion (Jiao et al., 2022), with lower *RMSD* values indicating better preservation of the enzyme’s 3D conformation (Dong et al., 2018; Wang R. et al., 2020). During the 50 ns simulation, the *RMSD* of the WT increased to 4.5 Å, whereas the *RMSD* of the mutant stabilized between 3.5–4.0 Å after 20 ns (Figure 3A). These findings suggested that the combination of EAHNWG with LA resulted in a more stable overall conformation, potentially accounting for the increased thermostability observed in the mutants (Jiao et al., 2022). Protein stability tends to be inversely correlated with structural flexibility (Yang et al., 2022). To assess the magnitude of fluctuations in individual residues and thereby reflect the flexibility in protein regions, *RMSF* was employed (Jiao et al., 2022). The mutant EAHNWG exhibited higher *RMSF* values in the catalytic active center (Thr440-Leu450), lid domain (Thr93-Ile100), and distal loop region (Gly625-Arg630) compared to the WT (Figure 3B). These regions displayed a higher degree of freedom of movement, which could potentially enhance enzyme activity (Pang et al., 2023). Simultaneously, *RMSF* values in other regions remained constant or were lower than those of the WT, contributing to increased overall structural stability in the system (Offman et al., 2011).

The *Rg* value serves as an indicator of protein structural compactness, with larger values suggesting looser structures (Chi et al., 2023). The average *Rg* values for WT and EAHNWG were 27.35 ± 0.18 and 27.09 ± 0.12 Å, respectively, indicating that mutants exhibit a tighter protein structure (Supplementary Table S3). Analyzing *Rg* fluctuations during MD simulations (Figure 3C) further supported this observation, as the *Rg* values for the mutant system were consistently lower than those for WT after the enzyme-substrate binding, underscoring the enhanced stability of the mutant complex. *SASA* values provide insights into protein hydrophobicity and structural compactness. The average *SASA* values of EAHNWG were reduced by 812.18 Å^2^ compared to WT (Supplementary Table S3), indicating stronger hydrophobicity and a more compact structure for EAHNWG (Rahman et al., 2021). Fluctuations in *SASA* values (Figure 3D) suggested that the binding of EAHNWG and LA significantly influenced their stability. Notably, *SASA* values of the WT complex were higher than those of the mutant at 10–50 ns, with more severe fluctuations. This demonstrated that mutation rendered the enzyme structure more compact, resulting in less variability in exposed and buried surface regions (Rahman et al., 2021).

Hydrogen bonds play a crucial role in non-covalent bonding interactions, with a higher number indicating stronger bonding (Xu et al., 2020). Changes in the number of hydrogen bonds during MD simulation of the two complex systems are depicted in Figures 3E, F. The WT complex exhibited the lowest hydrogen bond number and density, with the number of hydrogen bonds consistently between 1–2. In contrast, the number of hydrogen bonds in mutant EAHNWG varied from 1–5. The average intramolecular hydrogen bond numbers for WT and EAHNWG were 717 and 720, respectively (Supplementary Table S3), suggesting that mutation facilitates the formation of hydrogen bonds between LA and the enzyme, thereby enhancing the conformational stability of the complex system. In summary, the mutant EAHNWG exhibited more stable structures than WT. The binding of substrate and enzyme enhanced the overall conformational stability of the complex, and greater substrate-enzyme affinity led to tighter binding, resulting in changes in secondary structure. This aligned with our previous data, indicating an increased proportion of helical structures in the secondary structure of the mutant (Supplementary Table S2).

### 3.4 Substrate affinity analysis of wild-type and mutant EnLOX

To provide a more precise assessment of the binding pattern between LA and EnLOX, the MD simulation trajectory was thoroughly analyzed to calculate the binding energy using the MM/GBSA method. Energy decomposition revealed that electrostatic interaction was the primary contributor to the binding energy in both two complexes, followed by van der Waals energy, with non-polar solvation contributions ranking last (Table 3). The ΔG_bind_ values for LA with WT and EAHNWG were −52.39 ± 6.36 and −54.14 ± 2.83 kcal/mol, respectively. The binding free energy of LA to EAHNWG was lower than that to WT, indicating a stronger substrate binding affinity for the mutant (Chinnasamy et al., 2020). This finding corroborated our earlier experimental data, demonstrating that the *K*_*cat*_ */K*_*m*_ values of mutant EAHNWG exceeded those of WT (Table 2).

**TABLE 3:** Binding free energies and energy components predicted by MM/GBSA (kcal/mol).

Enzymes	ΔE_vdW_	ΔE_elec_	ΔG_GB_	ΔG_SA_	ΔG_bind_
EnLOX	−45.26 ± 1.39	−227.74 ± 8.81	227.76 ± 12.58	−7.16 ± 0.24	−52.39 ± 6.36
EAHNWG	−46.12 ± 1.86	−173.50 ± 9.66	172.54 ± 13.38	−7.09 ± 0.30	−54.14 ± 2.83

ΔE_vdW_: van der Waals energy; ΔE_elec_: electrostatic energy; ΔG_GB_: electrostatic contribution to solvation; ΔG_SA_: non-polar contribution to solvation; ΔG_bind_: binding free energy.

**FIGURE 4:**
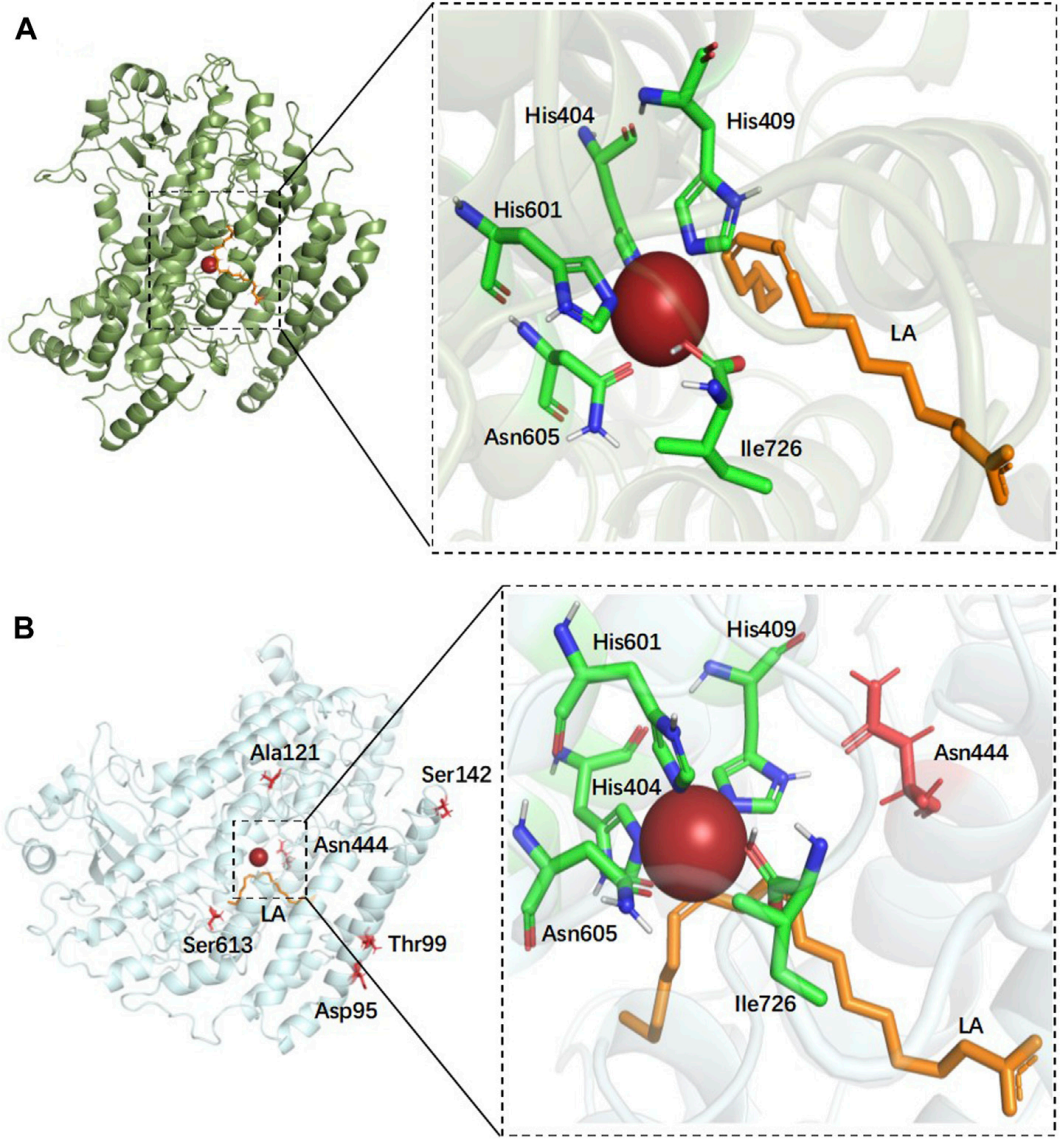
Docking results of wild-type EnLOX with LA and distribution of individual mutation residues in mutant EAHNWG. **(A)** The docking results of wild-type EnLOX and LA; **(B)** Distribution of the individual mutation residues in mutant EAHNWG. Positions of mutation residues (red), iron atom (red sphere), substrate LA (orange).

In order to gain insight into the catalytic process, the structure of EnLOX and its binding to the substrate LA were furtherly investigated. LA was docked into a protein-binding cavity centered on the catalytic active sites His404-His409-His601-Asn605-Ile726, as depicted in Figure 4A. The 3D structure of the enzyme with each mutation site in mutant EAHNWG was analyzed, as presented in Figure 4B. Site 444 was situated in proximity to the substrate-binding pocket and played a crucial role in substrate binding and catalysis. Sites 142, 95, and 99 were located within the lid domain of the enzyme, while site 121 was found in the N-terminal loop, and site 613 resides in the C-terminal loop.

**FIGURE 5:**
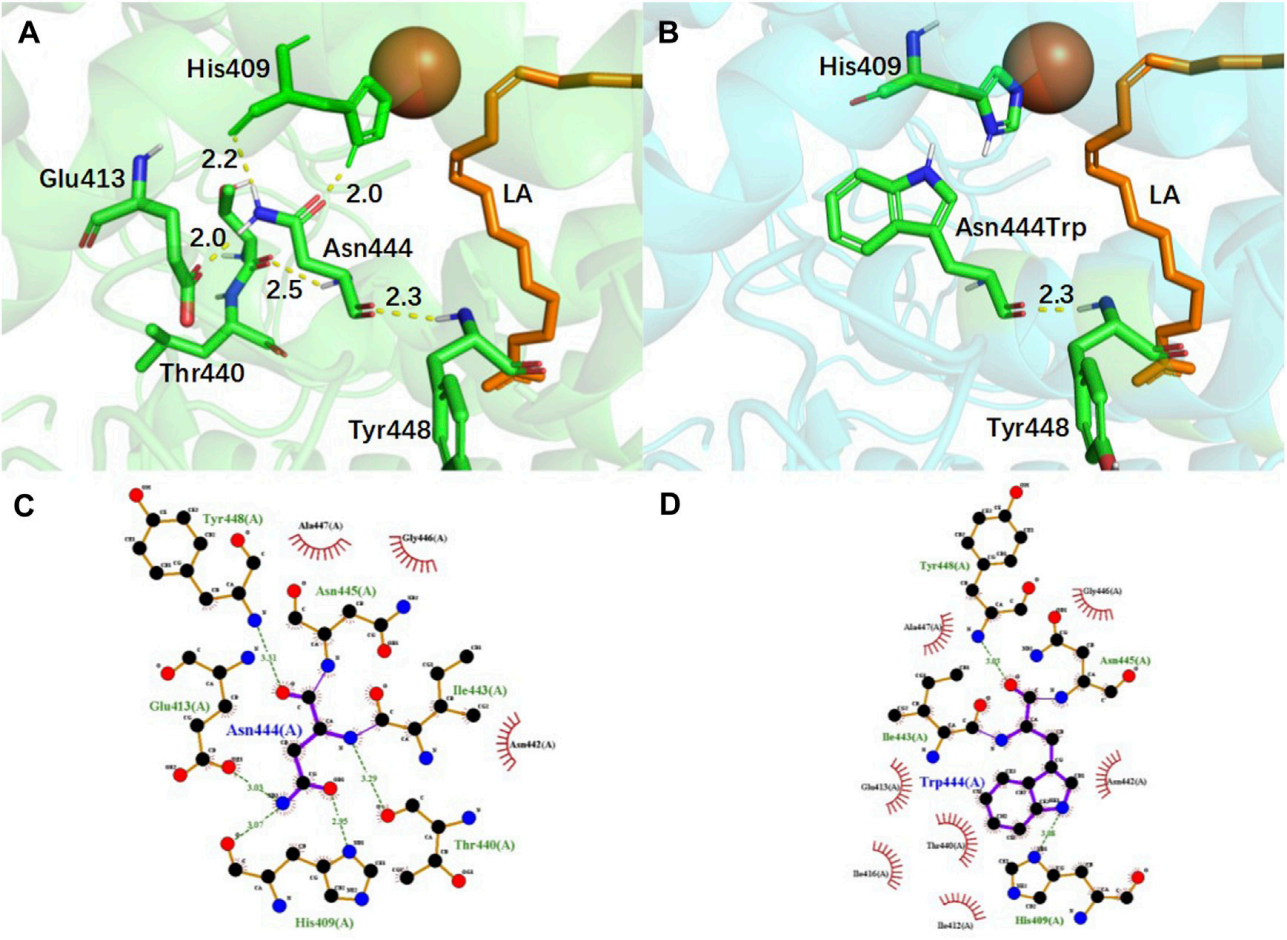
Conformational and hydrophobic interaction changes of EnLOX wild-type and its mutant EAHNWG. **(A,B)** Conformational changes of WT and mutant EAHNWG at residue 444; **(C,D)** Hydrophobic interactions changes of WT and mutant EAHNWG at residue 444. WT (green), mutant EAHNWG (blue), iron atom (red sphere), substrate LA (orange), hydrogen bond (yellow dashed line).

To examine the impact of mutations on the complex structure of EnLOX and LA, an analysis of the interaction forces around the mutation site was conducted, and the results are depicted in Figure 5. For the Asn444 site, five hydrogen bonds were formed with residues Glu413 (2.0 Å), His409 (2.0 Å, 2.2 Å), Thr440 (2.5 Å), and Tyr448 (2.3 Å) (Figure 5A). After the substitution of Asn with Trp, only one hydrogen bond was formed with residue Tyr448 (2.0 Å) (Figure 5B). Notably, the mutated amino acid residue Trp444 disrupted two hydrogen bonds with the catalytic key site His409, resulting in a more flexible α-helix formed by residues Thr440-Leu450. This enhanced flexibility facilitated substrate access to the catalytic center and product release, ultimately improving the catalytic activity of EnLOX (Pang et al., 2023). This finding aligned with previous results indicating that EAHNWG exhibited a higher *RMSF* value in Thr440-Leu450 compared to the wild-type. Furthermore, the reinforcement of hydrophobic interactions promoted reduction reactions in catalytic processes (Zhong et al., 2019). In the WT, residue Asn444 formed hydrophobic interactions with the surrounding residues Ala447, Gly446, and Asn442 (Figure 5C). However, Trp444 introduced four additional hydrophobic interactions with neighboring residues Gln413, Thr440, Ile416, and Ile412 (Figure 5D) after the mutation from the hydrophilic residue Asparagine to the hydrophobic residue Tryptophan. In the catalytic process of LOX, the excited state LOX binds to PUFA, leading to stereoselective dehydrogenation of the carbon atom in PUFA connecting the conjugated double bond—a rate-limiting step in the LOX reaction. Strengthened hydrophobic interactions promote the selective acquisition of hydrogen atoms from the substrated LA by EnLOX, thereby reducing the duration of this rate-limiting step and increasing its catalytic activity (Egmond et al., 1973; Wu and Wang, 1998).

**FIGURE 6:**
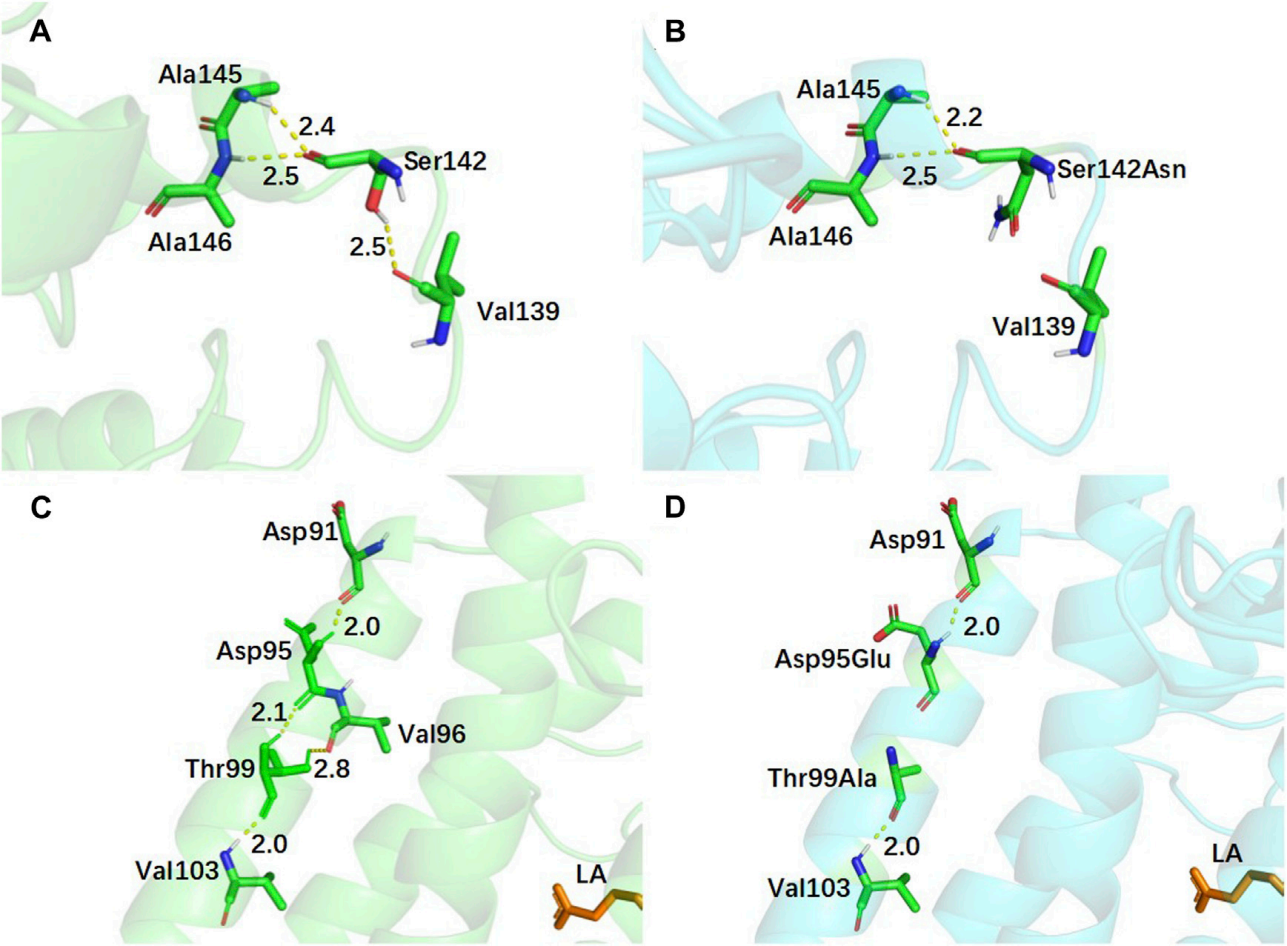
Conformational changes of EnLOX wild-type and its mutant EAHNWG. **(A,B)** Conformational changes of WT and mutant EAHNWG at residue 142; **(C,D)** Conformational changes of WT and mutant EAHNWG at residues 95 and 99. WT (green), mutant EAHNWG (blue), hydrogen bond (yellow dashed line).

In addition to residues located near the substrate binding pocket, mutations in the lid domain of EnLOX may also contribute to its enhanced catalytic activity. The lid, comprising residues Leu59-Leu202, plays a crucial role in EnLOX function. Specifically, residue Ser142 is situated within the loop connecting two α-helices in the lid domain (Figure 6A). The mutation from Ser142 to Asn142 resulted in the loss of a hydrogen bond between this residue and Val139 (Figure 6B). This loss enhanced the flexibility of the loop (Thr109-Ile144) in the lid domain, thereby facilitating the opening and closing of the lid (Pang et al., 2023). This increased flexibility allowed for easier access of the substrate LA to the catalytic domain, ultimately enhancing catalytic efficiency. Changes in the number of hydrogen bonds at specific amino acid sites within the mutant can also affect the regional flexibility of the mutant enzyme (Chi et al., 2023). After replacing Asp95 and Thr99, located on the α-helix of the lid domain, with Glu95 and Ala99, the total number of hydrogen bonds at these two sites decreased from 5 to 3. Furthermore, the two hydrogen bonds between Asp95, Thr99, and Val96 disappeared (Figures 6C, D). This reduction in the number of hydrogen bonds increased the flexibility of the lid domain, promoting the adsorption of substrates and facilitating their entry into the catalytic domain (Pang et al., 2023). Sites 122 and 613, which are not directly interacting with the active sites due to their distance from the catalytic pocket (Figure 4B), were expected to exert a distal effect resulting from the introduction of mutations (Jiménez-Osés et al., 2014). This effect indirectly influenced enzyme-substrate binding or the conformational state of the enzyme, ultimately leading to changes in the catalytic efficiency and enzymatic properties of EnLOX (Zhou Y. et al., 2022).

## 4 Discussion

Commercial LOX is primarily acquired through the extraction of plant tissues (Hui et al., 2017). However, industrial-scale production of LOX faces significant challenges stemming from low extraction rates and enzyme purity (Diao et al., 2016). Microbial heterologous expression has been employed to overcome these limitations, yet it still presents drawbacks such as low catalytic efficiency and poor stability, rendering it unsuitable for industrial applications (Zhang et al., 2022). Hence, it becomes imperative to undertake modifications aimed at obtaining LOX mutants characterized by enhanced catalytic activity and improved thermostability. This endeavor holds significant importance for facilitating the widespread industrial utilization of LOX.

Directed evolution stands out as one of the most favored protein engineering techniques for enhancing enzyme catalytic activity. This method permits the introduction of a multitude of random mutations based solely on amino acid sequence information, offering a broad spectrum of possibilities to augment enzyme performance. Presently reported LOX enzymes suffer from inherent drawbacks such as low catalytic efficiency, poor thermal stability, and inadequate substrate specificity. These limitations fall short of meeting the requirements for industrial applications (Zhang et al., 2022). Directed evolution represents an effective avenue for improving enzyme catalytic activity. This method involves the deliberate creation of conditions mirroring natural enzyme evolution, followed by high-throughput screening to identify mutants with enhanced catalytic capabilities (Larissa et al., 2012). Commonly employed techniques include *error-prone* PCR and *DNA shuffling* (Wang H. Y. et al., 2020; Xiong et al., 2021). Numerous enzymes have undergone enhancement of catalytic activity through directed evolution strategies (Jiao et al., 2022). For example, Besirlioglu achieved a phytase mutant enzyme with a remarkable 79 U/mg increase in specific viability using directed evolution and high-throughput screening techniques (Besirlioglu et al., 2017). Pan employed directed evolution to obtain Asn329, a salinophilic α-amylase with a remarkable 14.6-fold increase in enzyme activity (Pan et al., 2020). To date, there is only one documented instance of directed evolution applied to LOX. Guo successfully obtained the mutase D22 of *Anabaena* LOX through directed evolution, leading to a slight increase in thermal stability and catalytic activity (Guo et al., 2014). Thus far, LOX mutants with both high catalytic activity and thermostability by directed evolution have remained elusive for industrial applications.

Enzymes that find successful industrial applications possess specific characteristics, namely, the ability to maintain robust catalytic activity and stability across a broad spectrum of pH and temperature ranges. Only those enzymes meeting these stringent criteria can be deemed potential candidates for novel food applications. It is worth noting that the properties of lipoxygenases (LOXs) can vary significantly depending on their source. LOXs derived from *Anabaena* and *P*. *aeruginosa*, for instance, exhibit low catalytic activity, limited pH stability, and thermostability challenges (Kalms et al., 2017; Qian et al., 2018). Qian et al. identified a LOX from *Myxococcus xanthus* that performed well under acidic conditions (pH 3.0–6.0), but it displayed poor thermal stability (*t*_*1/2*, 50°C_ = 7.1 min) and lost its catalytic activity after a mere 15 min at 65°C (Qian et al., 2017). While LOXs from *Pleurotus* and *A*. *aegerita* exhibited higher catalytic activity, their optimal reaction temperatures (20°C–35°C) present limitations for industrial use (Kuribayashi et al., 2002; Karrer and Ruhl, 2019). In this study, a mutant EnLOX denoted as EAHNWG, featuring six residue mutations, was obtained through a directed evolutionary strategy. Its specific activity reached 330.17 ± 18.54 U/mg, marking an impressive 8.25-fold increase compared to the wild-type EnLOX. Notably, while the optimal reaction pH for mutant EAHNWG remained the same as that of the wild-type, its residual activity after 10 h of incubation at pH levels ranging from 6.0 to 11.0 surpassed that of the wild-type. Consequently, the mutant demonstrated heightened stability in mildly acidic and alkaline environments, rendering it suitable for complex food processing conditions. Furthermore, the mutant EAHNWG displayed a *t*_*1/2*_ value of 6.44 ± 0.24 h at 50°C, representing a 0.4-h improvement compared to the wild-type (6.04 ± 0.14 h). This enhancement in thermal stability positioned the mutant as a promising candidate for industrial applications. Importantly, the *K*_*m*_ values of the mutant EAHNWG, as determined in this study, were lower than those of LOXs derived from *Anabaena* (3.54 mM) (Guo et al., 2014), *Calothrix* (6.70 μM) (Qi et al., 2020) and *P. aeruginosa* (6.40 mM) (Pang et al., 2023). Furthermore, its *K*_*cat*_ */K*_*m*_ value significantly outperformed those of *M. xanthus* LOX (0.02/(s μM)) (An et al., 2018), *Pleurotus ostreatus* LOX (0.2/(s μM)) (Kuribayashi et al., 2002) and *Rivularia* LOX (10.79/(s μM)) (Qi et al., 2020), closely with *Calothrix* LOX (13.85/(s μM)) (Qi et al., 2020). The enhanced thermal stability, catalytic efficiency, and improved substrate affinity collectively positioned the mutant EAHNWG as a versatile asset in the food industry’s arsenal.

To delve into the molecular mechanism underlying the increase in catalytic activity of EnLOX due to key amino acid mutations, researchers conducted an analysis of force alterations through MD simulation and protein 3D structure analysis (Childers and Daggett, 2017). Comparative analysis with the wild-type enzyme revealed several noteworthy changes in the mutant EAHNWG. Firstly, the mutant displayed an increased number of intramolecular hydrogen bonds and exhibited decreased values for *RMSD*, *SASA*, and *Rg* when compared to the wild-type. These observations implied the enhanced stability within the mutant enzyme-substrate complex, providing an explanation for the improved thermal stability of EAHNWG. Furthermore, the residues associated with the catalytic activity center of EAHNWG exhibited higher *RMSF* values compared to the wild-type, indicative of increased flexibility in this crucial region, which effectively enhanced the catalytic activity of EnLOX. It is worth noting that certain beneficial mutations can impact key amino acids within the enzyme’s catalytic activity center, subsequently increasing the binding free energy between the enzyme and substrate, thereby elevating catalytic activity (Xie et al., 2021). In the case of mutant EAHNWG, there was a reduction in the binding energy with the substrate LA, suggesting enhanced substrate binding strength, a favorable trait for catalytic activity. Catalytic activity and stability in enzymes are influenced by a multitude of factors, including hydrogen bonds, hydrophobic interactions, electrostatic interactions, substrate channel size, and amino acid composition (Wu et al., 2017; Chi et al., 2023). Alterations in the number of hydrogen bonds near the catalytic pocket and around the active sites can induce changes in the hydrogen bonding network, consequently impacting the flexibility of the catalytic region and thus affecting catalytic activity (Zhou J. et al., 2022). Specifically, in mutant EAHNWG, the loss of four hydrogen bonds involving Trp444 and residues Glu413, His409, and Thr440 within the substrate-binding pocket contributed to increased flexibility in the α-helical region (Thr440-Leu450), facilitating improved substrate access to the catalytic pocket. In previous research, alterations in the hydrogen bond configuration at the entrance to the substrate pocket of olive LOX has been found to reduce spatial hindrance in this region, leading to a remarkable 93-fold increase in catalytic activity (Palmieri-Thiers et al., 2011). Furthermore, hydrophobic amino acids played a crucial role during the catalytic process of LOX, as they contribute to stabilizing the binding of the substrate, thus influencing enzyme activity (Miled et al., 2003). Enhanced hydrophobic interactions can facilitate the deprotonation of carbon atoms in small molecules (Zhong et al., 2019; Mokhtar et al., 2020). The hydrophobic interaction involving the mutant EAHNWG at Trp444 was notably intensified. This enhancement could induce the enzyme to selectively obtain hydrogen atoms from LA, thereby advancing the reduction step in the catalytic reaction (Egmond et al., 1973; Wu and Wang, 1998). It is worth noting that the mutation of aspartate at position 305 in *Anabaena* LOX improved the enzyme’s thermostability and catalytic activity, a finding that bears similarities to our results (Guo et al., 2014). The heightened hydrophobic effect observed in *Anabaena* LOX near the mutation site promoted enhanced catalytic activity and thermostability of the enzyme, aligning with our own findings (Qian et al., 2018). Interestingly, although the lid domain was not directly involved in the substrate binding site, it covered the catalytic pocket and regulated the interaction between the substrate and the active sites, thereby impacting catalytic activity (Iqbal et al., 2017). The structural domain of the lid of LOX has a degree of motion flexibility. Pang et al. converted Lys138-Ala144 of *P. aeruginosa* LOX into a highly flexible loop. The mutation reduced the spatial resistance of the substrate-binding region and increased the flexibility of the lid structure, which improved the likelihood of substrate binding to the catalytic domains, thereby increased catalytic activity (Pang et al., 2023). Mutations at sites 142, 95, and 99 in this study resulted in a reduction in the number of hydrogen bonds within the lid domain and increased the flexibility of the lid, consequently enhancing catalytic activity (Pang et al., 2023).

In all, a mutant enzyme, EAHNWG, was generated through directed evolution approach, resulting in an impressive 8.25-fold increase in catalytic activity. Molecular dynamics (MD) simulations revealed that the complex of mutant EAHNWG and substrate LA exhibited enhanced structural stability. Furthermore, the analysis of 3D structure highlighted several key factors contributing to the heightened activity. Specifically, these factors included a reduction in the number of hydrogen bonds within the catalytic pocket, an augmentation of hydrophobic interactions, increased flexibility of the lid domain, and a stronger binding affinity between the enzyme and substrate. These findings advanced our comprehension of the structural and functional interplay within LOX enzymes, and provided valuable insights for the targeted modification of LOX catalytic activity. Importantly, mutant EnLOX, characterized by improved catalytic efficiency and stability, holded great promise for various industrial applications.

## Data Availability

The datasets presented in this study can be found in online repositories. The names of the repository/repositories and accession number(s) can be found below: https://www.ncbi.nlm.nih.gov/, protein/WP_074927588.1.
