# Supplementary material for: Improved catalytic performance and molecular insight for lipoxygenase from Enterovibrio norvegicus via directed evolution
Source: Front Bioeng Biotechnol. 2023 Nov 16;11:1305582. doi: 10.3389/fbioe.2023.1305582 (PMC10690365; doi:10.3389/fbioe.2023.1305582)
Supplement: Supplementary file 1 [file Table1.DOCX]

Supplementary Material

# Supplementary Figures and Tables

## Supplementary Tables

**Supplementary Table 1.** Biochemical properties of EnLOX and LOXs from various sources.

| **Enzymes** | **Specific activity (U mg^-1^)** | ***K_m_* (μmol L^-1^)** | ***K_cat_* (s^-1^)** | ***K_cat_/K_m_* (L μmol^-1^ s^-1^)** | **Reference** |
| --- | --- | --- | --- | --- | --- |
| *E. norvegicus* LOX | 40.02 | 3.49 | 16.86 | 4.83 | (Zhang et al., 2022) |
| *Calothrix* LOX | 73.10 | 6.7 | 93.2 | 13.85 | (Qi et al., 2020) |
| *Rivularia* LOX | 68.8 | 7.5 | 80.6 | 10.79 | (Qi et al., 2020) |
| *T. bouteillei* LOX | 10.10 | 16.5 | 12.8 | 0.78 | (Qi et al., 2020) |
| *Anabaena* LOX | 10.40 | 56 | 30.3 | 0.54 | (Zhang et al., 2012; Diao et al., 2016) |
| *M. xanthus* LOX | 24.00 | 380 | 9.2 | 0.02 | (Hui et al., 2017 , An et al., 2018) |
| *P. aeruginosa* LOX | 28.50 | 48.9 | 23.5 | 0.48 | (Lu et al., 2013) |
| *B. thailandensis* LOX | 26.40 | 41.5 | 93.7 | 2.26 | (An et al., 2015) |
| *A. aegerita* LOX | 51.34 | 295.5 | 103.9 | 0.35 | (Karrer and Ruhl, 2019) |
| *N.benthamiana* LOX | 0.35 | 3.9 | NR | NR | (Schwab, 2011) |
| *Pleurotus* LOX | 130.30 | 40.3 | 157 | 3.89 | (Kelle et al., 2014) |
| *Cyanothece* LOX | NR | 18 | 30.7 | 1.7 | (Alexandra et al., 2010) |
| *P. ostreatus* LOX | 33.00 | 130 | 25.7 | 0.2 | (Kuribayashi et al., 2002) |

**Supplementary Table 2.** Proportions of secondary structures in the wild-type and mutant EAHNWG.

| **Secondary structure** | **Proportion / %** | |
| --- | --- | --- |
|  | **WT** | **EAHNWG** |
| α-helices | 26.9 | 34.6 |
| Strand | 17.9 | 11.7 |
| Turns | 20.9 | 23.9 |
| Random coli | 34.3 | 29.8 |

**Supplementary Table 3.** Hydrogen bonds number, *Rg*, and *SASA* values for WT and its mutant EAHNWG

| **Enzymes** | **Hydrogen Bonds Number** | ***Rg* (Å)** | ***SASA* (Å^2^)** |
| --- | --- | --- | --- |
| WT | 717±0.00 | 27.35±0.18 | 35586.87±821.57 |
| EAHNWG | 758±0.39 | 27.09±0.12 | 34774.69±748.37 |
